# MALDI-TOF MS experience with the identification of complex microorganisms using two devices in a National Reference Laboratory

**DOI:** 10.1128/spectrum.01677-24

**Published:** 2025-03-17

**Authors:** Maria Florencia Rocca, Diego Danze, Gastón D´Angiolo, Paula Etcheverry, Claudia Martínez, Mónica Prieto

**Affiliations:** 1 Servicio Bacteriología Especial, Instituto Nacional de Enfermedades Infecciosas, ANLIS Dr. Carlos G. Malbrán, CABA, Argentina; 2 Red Nacional de Espectrometría de Masas aplicada a la Microbiología Clínica ReNaEM, Buenos Aires, Argentina; Icahn School of Medicine at Mount Sinai, New York, New York, USA

**Keywords:** MALDI-TOF, mass spectrometry, verification, unusual pathogen, RENAEM, network

## Abstract

Matrix-assisted laser desorption/ionization time-of-flight (MALDI-TOF) methodology has meant a true revolution in clinical microbiology, optimizing diagnosis times through reliable results in a matter of minutes, replacing phenotypic and molecular procedures in several cases. Today, two major platforms are commercially available, the MALDI Biotyper (Bruker Daltonics Inc., Bremen, Germany) and the VITEKMS PRIME (bioMérieux, Marcy l'Etoile, France). In Argentina, the pioneering adoption of this technology took place at the National Reference Laboratory of the National Institute of Infectious Diseases, then a strategic network has emerged to disseminate the experience to all clinical laboratories utilizing MALDI-TOF and oversee their operational efficacy. In this study, we developed a strictly in parallel comparative evaluation of the performance of the Microflex LT and the next generation VITEK MS PRIME for the identification of a large number of complex microorganisms with clinical relevance, based on updated platforms and databases. The overall agreement of correct identifications of both systems was 98.23%, with the same or better resolution as rRNA gene sequencing but with cheaper and faster. Each has its advantages and limitations, so it will be up to each individual laboratory to evaluate which one is most appropriate for its workflow. Once purchased, both platforms represent enormous cost savings in supplies and labor in any laboratory.

**IMPORTANCE:**

This work detailed a matrix-assisted laser desorption/ionization time-of-flight (MALDI-TOF) experience with the identification of a large set of complex microorganisms using two devices in the settings of a National Reference Laboratory. In Argentina, we coordinate the national network of mass spectrometry (MS), an extended work group formed in year 2015 that includes all public and private laboratories that have incorporated the technology as a diagnostic tool through the different commercial platforms available on the market. At this time, as a National Reference Laboratory, we conducted this comprehensive assessment of MALDI-TOF MS performance for the identification of diverse bacteria, specifically targeting pathogens of significant public health concern. During investigation, we identified certain limitations inherent to the methodology and the results obtained have considerable impact that must be transferred to the users of MS platforms in clinical laboratories all over the world.

## INTRODUCTION

Mass spectrometry (MS) through the MALDI-TOF (matrix-assisted laser desorption/ionization time-of-flight) methodology has meant a true revolution in clinical microbiology, optimizing diagnosis times through reliable results in a matter of minutes, replacing phenotypic and molecular procedures in several cases (1).

Today, two major MALDI-TOF MS platforms are commercially available, the MALDI Biotyper (Bruker Daltonics Inc., Bremen, Germany) and the VITEKMS (bioMérieux, Marcy l’Etoile, France).

Both MS systems consist of mass spectrometer hardware, licensed software, reference spectrum databases, and different sample preparation and protein extraction methods. While numerical scores or confidence results between the systems are not directly comparable due to proprietary algorithms and databases, comparison studies have shown similar high performance for the identification of common bacteria (2–5).

In 2013, both MALDI-TOF MS systems were approved for *in vitro* diagnostics by the Food and Drug Administration for the identification of microorganisms cultured from human specimens. The scarce differences noted do not impact the intended use and do not raise questions as to the safety and effectiveness of the devices. Briefly, the samples are submitted to multiple laser shots inside the equipment. The matrix absorbs the laser light and vaporizes, along with the sample, gaining an electrical charge (ionization) in the process. Electric fields then guide the ions into a vacuum tube, which separates them according to their mass, with the smaller molecules traveling through the flight tube faster than the larger molecules. This “time of flight” creates a series of peaks corresponding to the different molecules contained in the organism from the sample. All of these peaks create a spectrum that is unique for that microorganism. The precise species can be identified quickly and easily by comparing the spectrum to a vast comprehensive library owned by each manufacturer (6, 7).

In Argentina, the pioneering adoption of MALDI-TOF MS technology took place at the National Reference Laboratory of the National Institute of Infectious Diseases and the Clinical Bacteriology Laboratory of the Teaching Hospital at the Faculty of Pharmacy and Biochemistry, University of Buenos Aires. These institutions, serving as reference laboratories in bacteriology and mycology, boast extensive culture collections encompassing a diverse array of bacterial and fungal species meticulously characterized through polyphasic taxonomy methodologies.

From a public health perspective, a strategic network has emerged to disseminate the experience results to all clinical laboratories utilizing MALDI-TOF MS and oversee their operational efficacy. This extended work group formed in 2015 includes all public and private laboratories that have incorporated the technology as a diagnostic tool through the different commercial platforms available on the market (8). At that time, as a National Reference Laboratory, we conducted a comprehensive assessment of MALDI-TOF MS performance for the identification of diverse bacteria and fungi, specifically targeting pathogens of significant public health concern in our country. During these investigations, we identified certain limitations inherent to the methodology (9), which has a performance similar to sequencing the 16S rRNA gene and does not allow discrimination of closely related species; then, detailed information on more than 2,000 microorganisms has been collected in a manual for interpreting MALDI-TOF results, with alternatives for the identification of rare or unusual bacteria, which are not completely resolved by this technique.

So, one of the purposes of this network is the development and transference of *in-house* databases and the verification of new libraries for the identification of different taxonomic groups to be able to transmit unified reporting criteria to less complex laboratories (10–13).

Other investigators have studied the use of MS for specific groups of bacteria (14–16); but in this study, we developed a strictly in parallel comparative evaluation of the performance of the Microflex LT and the next-generation VITEK MS PRIME for the identification of a large number of complex microorganisms with clinical relevance, based on updated platforms and databases in our reference institution.

Our objective was to evaluate the performance of the new VITEK MS PRIME system through the IVD V3.2 and SARAMIS RUO libraries v4.17 and the Microflex LT system, with its MBT Compass Explorer v12 library, for the identification of a large number of complex microorganisms that require molecular methods for their complete characterization in the settings of the National Reference Laboratory. And then, the advantages and limitations of each one with respect to the different bacterial taxonomic groups tested will be discovered.

## MATERIALS AND METHODS

A total of 882 bacterial isolates were processed from our culture collection belonging to the Culture Collections Federation for Latin America and from other laboratories that have collaborated with their characterized strains.

The bacterial isolates were frozen at −80°C in trypticase soy broth with 10% glycerol.

The laboratory routine was also processed prospectively for 6 months to compare the performance of both platforms.

We grouped the isolates into families according to their taxonomy (Table 1).

**TABLE 1:** The bacterial isolates used in this research were separated in the following taxonomic groups

Taxonomic group	Number of isolates
*Actinomycetales*	70
*Aeromonas and Vibrio*	22
*Fastidious gram-negative bacilli*	76
*Sporulating ram-positive bacilli (GPB*)	29
*Non-sporulating GPB*	55
*Coryneform GPB*	60
*Non-fermenting gram-negative bacilli*	225
*Catalase-negative gram-positive cocci (GPC*)	173
*Catalase-positive GPC*	48
*Enterobacterales*	124
Total	882

### Culture conditions

The culture of the strains was carried out on trypticase soy agar supplemented with 5% sheep blood, and the incubation conditions were 18–24 h, 37°C in aerobic conditions or up to 48 h in a 5% CO_2_ atmosphere in the case of slow-growing bacteria. This was repeated throughout all the evaluation projects to maintain standardized conditions. However, it is known that a wide variety of culture media were already tested for both platforms and with high-confidence identifications (17).

The gold standard method used for the reference identification of all isolates was polyphasic taxonomy: constitutive gene amplification and sequencing for the different families according to the CLSI MM18 standard guidelines (18), in addition to biochemical and serological tests. Sequencing and amplification of *16S rRNA* gene were carried out using the primers corresponding to the position 8-27F (5′-AGAGTTTGATYMTGGCTCAG-3′) and 1492R (5′-ACCTTGTTACGACTT-3′) of the 16S *rRNA* gene of *Escherichia coli* as it has been described (18, 19).

In the case of complex taxonomic groups, the confirmation of the identification was done using specific primers; for example, *recA* gene for the isolates belonging to the *Burkholderia cepacia* complex (20); *rpo*B gene for *Corynebacterium* species (21) and *secA* gene on the *Actinomycetales* isolates (22).

PCR products were sequenced using the Big Dye Terminator v3.1 Cycle sequencing kit equipment (Applied Biosystems, San Francisco, EEUU) and analyzed in the ABI 377 Genetic Analyzer (Applied Biosystems).

The sequences obtained were compared with standard sequences deposited in the NCBI Gene Bank (National Center for Biotechnology Information; http://www.ncbi.nlm.nih.gov/genbank), using the BLAST V 2.0 software (Blast Internet Services, Pittsboro, NC, USA) and interpreted according to CLSI standards.

Procedure on MALDI-TOF MS platforms. On the same day, the same operator spotted the isolate on the plate in duplicate, using the *in-situ* extraction method, with some novel modifications as described below:

For VITEK MS PRIME, the seeding method consisted of adding 0.5 µL of 100% formic acid (FA) in each spot of the disposable slide, then, a portion of an isolated colony in its exponential growth phase was collected with a wooden stick, validated by the lab for several years, or using the VITEK PICKME pen, acquired later, depending on the operator, and mixed in the well with the FA. Once dried, the spot was covered with 1 µL of commercial matrix CHCA (α-cyano-4-hydroxycinnamic acid) immediately or at most per acquisition group.

For MICROFLEX LT, 1 µL of 100% FA was added to each spot of the steel plate, then the colony was added, using the wooden stick as normally routine at the laboratory, allowed to dry, and CHCA was added within 30 min after application.

Based on the great diversity of isolates, for standardization, all isolates were seeded with FA despite the preparation suggested of the manufacturers.

Precisely, the majority of clinically bacteria have cell walls. The thickness of those cells walls, as well other components, can affect identification rates using MALDI-TOF MS which can be improved using additional extractions steps, such as the addition of FA.

### Databases

The acquired spectrum was analyzed by pattern matching with the default settings in MBT Compass Explorer Bruker library v.12 and bioMérieux IVD v3.2, which were the latest updates available at the time of the study. All the non-identified (NI) in the IVD database were compared to the super spectra and reference spectra in the SARAMIS v4.17 databases of the RUO mode to evaluate any improvement in the identifications.

The MBT Compass reference library v12 covers 4,274 species of 704 microorganism genera. This database used is for research use only (RUO), and the software module also offers multiple sophisticated bioinformatics algorithms for spectrum comparison and statistical data interpretation, enabling users to optimize the microorganism classification process by creating, modifying and organizing data and *in-house* libraries.

IVD V3.2 Knowledge Base comprises the closed taxon database for IVD use, developed by bioMérieux. It is created from 47,204 spectra of 15,556 strains, covering 1,316 species (1,095 bacterial and 221 fungi).

SARAMIS KB V4.17—Research Use corresponds to the taxon database for RUO. It is created from 37,043 reference spectra and 4,929 super spectra, covering a total of 2,286 taxa (including bacteria, mycobacteria, filamentous fungi, yeasts, and algae) and also allows the creation of super spectra and analysis of biomarker peaks by the user.

The corresponding calibration procedure was carried out before each run, and the identification criteria were according to the recommendations of each manufacturer as described below.

The *E.coli* ATCC 8739 strain for the calibration on VITEK MS PRIME, should be incubated for 18–24 h at 35 ± 2°C on blood agar under aerobic atmosphere. The strain has to be deposited on positions: xA1, xB1, and xC1, depending on the number of samples tested (one calibrant per acquisition group of 16 spots). Using a loop, cells of *E. coli* ATCC 8739 were spotted on the calibration position of each acquisition group on the target slide. Immediately, 1 µL of VITEK MS-CHCA matrix was added to each calibration spot using a pipette. Each spot was allowed to dry completely.

For Microflex LT, the platform was calibrated prior to each assay using the BTS bacterial test standard (Bruker Daltonics, Bremen, Germany) which consists of a commercial extract of ribosomal proteins from a standard strain of *E. coli* DH5-α supplemented with two additional proteins (RNAse and myoglobin), used for the detection of standard peaks in the range of 3,637–16,952 Da within a tolerance range for the position of each peak, as recommended by the manufacturer (Flex control 3.4 user manual, 2011).

### Identification criteria

Briefly, mass spectra that result in a score value ≥2.0 are considered high-confidence identifications in MICROFLEX LT. If the first and second best matches yield different species but belong to the same genus, they are considered low-confidence identifications. Mass spectra that provide a score value between 1.7 and 1.99 are considered identifications at the genus level. Values < 1.70 are reported as NI.

For VITEK MS PRIME, spectra that provide confidence values of 60–99.9% for similarity to a reference species in the database are considered high-confidence identifications. Identifications are defined as low discrimination when a spectrum equally matches two, three, or four species. No identification is reported when there is no match in the database or when there are more than four matching species, resulting in a low-confidence value.

## RESULTS

For protein extraction, it is well known that direct sample spotting is the simplest approach; however, for bacteria with cell walls, mucoid or pigmented colonies, this technique could give lower identification rates, as described by van Veen et al. (15). The *in-situ* extraction method with FA is very simple, resulting in good resolution to species level in most cases; that is why we decided to apply this protocol with minor modifications.

On the other hand, the well-known tube extraction method is more complex and takes more time; we find it suitable for inactivation of highly pathogenic bacteria (23) and for library creation. So, we did not perform it in the study; besides, the protocols are different for each platform which would make it difficult to standardize the procedure in both MS systems.

In some cases, we used the VITEK PICKME pen for spotting the strains on the VITEK MS PRIME slide, which speeded up the work and standardized the biomass that was deposited in the well, yielding very good results in all identifications, prioritizing the correct calibration. This tool would be helpful for an operator who is not very experienced with direct preparation.

### Turnaround times

Sample plate preparation is very similar, but the overall workflow to obtain a final result is shorter in MICROFLEX LT (2–3 min vs 6–7 min) since the results appear individually per well read, unlike VMSP, where they appear once the entire quadrant has been analyzed, making it more suitable for processing larger batches of samples.

However, VITEK MS PRIME offers simultaneous loading of up to 16 slides that can be prepared by different operators or laboratories, which reduces its longer response times and makes it appropriate for laboratories with fewer identifications in general but with more stations of work in parallel. In addition, VITEK MS PRIME offers the option to prioritize a slide as urgent so it is processed before the others and this new version allows an automatic fine tuning.

### Reagents and calibrators

The Bruker matrix CHCA and BTS calibrator must be rehydrated in organic solvent (50% acetonitrile + 47.5% H_2_O + 2.5% trifluoroacetic acid), while the VITEK MS system includes ready-to-use reagents.

On the other hand, despite the fact that Bruker now offers disposable plates, we had at the time of the study only the reusable 96-well plates, therefore a cleaning procedure with trifluoroacetic acid must be considered when all the wells are completed; while the bioMérieux slides are always disposable and each includes three acquisition groups with 16 spots each for a total of 48 wells to use; however, when one or more wells from an acquisition group is used, the empty wells cannot be used at a later time.

Regarding the calibrators, commercial lyophilized BTS has some requirements and storage recommendations, but certainly, it is easier to use than the *E. coli* standard strain ATCC 8739 used in VITEK MS PRIME; the biggest limitation that we found was when there is a failure in the calibration of the *E. coli* strain, which involves reseeding and reacquiring all the wells in that acquisition group.

### Global identification results

Because the expression of results is quite different between platforms, the identifications were considered correct at the species level when the library returned species that should be reported as groups or complexes due to the current taxonomic status, for example, species of the *Aeromonas caviae* complex (24), from the *Streptococcus mitis* group (25), or species of the *Enterobacter cloacae* complex (26), were analyzed as species/group or complex level (Table 2).

**TABLE 2:** Global identification performances of VITEK MS PRIME and Microflex LT commercial libraries for the total of microorganisms tested

Correct identifications	VITEK MS PRIME	Microflex LT
Species/complex or group	807 (91%)	766 (87%)
Genus	35 (4%)	91 (10%)
No identification (NI)^*a*^	40 (5%)	25 (3%)
NI with correct ID in RUO library^*b*^	21/40 (53%)	N/A^*c*^
Total	882	882

^*a*^ NI: Good quality of spectrum, correct calibration, but No confidence results.

^*b*^ NI comparing with IVD library, then were compared with the reference spectrum in RUO database of VITEK MS PRIME for probable improvements.

^*c*^ N/A, not applied.

The overall agreement of correct identifications at genus and species levels of both MS systems was 98.23%, calculated with https://calculator.academy/percent-agreement-calculator/.

**Fig 1:**
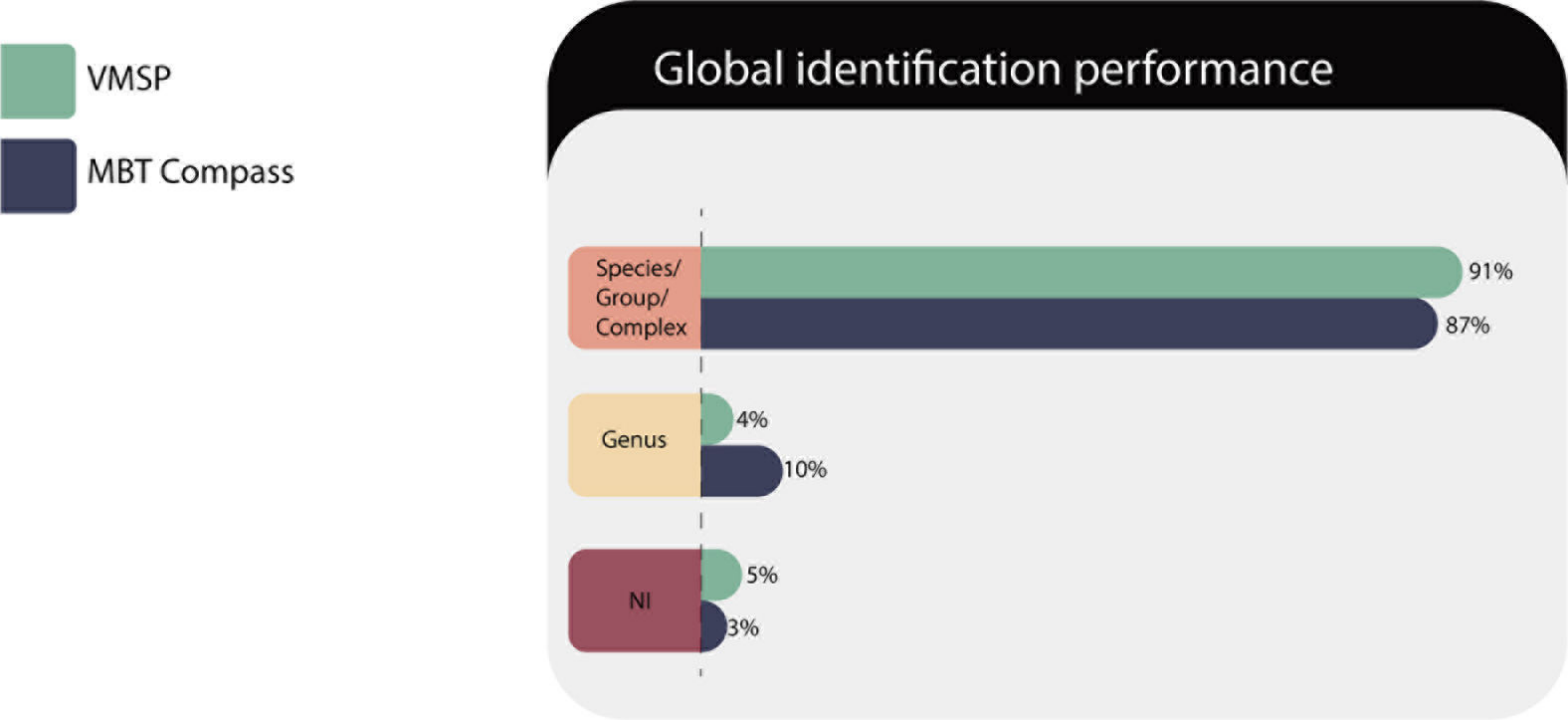
Global identification performances of both platforms over the total of isolates tested.

As shown in Fig. 1, correct species/complex or group identification was 91% for VITEK MS PRIME and 87% for Microflex LT; but considering correct identifications at least at the genus level, both platforms achieved 95% and 97%, respectively. These results represent important clinical impact in the case of microorganisms that are very difficult to identify in the short term.

The NIs were 3% and 5%, respectively, however, if we compare the NIs from IVD with the RUO database, this percentage on VITEK MS PRIME significantly reduced to 2.5%.

### Retests

Both platforms required repetitions in around 10% of the cases; for VITEK MS PRIME, they were due to failures in the calibration process with the *E. coli* strain and no identification results; while the repetitions in Bruker’s platform were due to low score values and bad spectrum without enough peaks.

It is worth clarifying that the bad spectra with low-confidence identifications were retested on both platforms, in the case of PRIME using the PICKME PEN and the final results are shown in this work.

There were no incorrect identifications in any case and most disagreements at the species level between platforms occurred in *Streptococcus bovis* group, *A.caviae* and *Aeromonas hydrophila*, *Achromobacter xylosoxidans* group, *Burkholderia* sp., *Pandoraea pnomenusa* and *Pandoraea sputorum*, and some *Corynebacterium* species, but we did not detect discrepancies at the genus level.

For the analysis of the results, the microorganisms evaluated were grouped as follows:

Gram-positive bacilli (GPB) comprising the *Actinomycetales* (70), sporulated GPB (29), non-sporulating GPB (55), and coryneforms (60).

Non-fermenting gram-negative bacilli (NFGNB, 225).

Fastidious gram-negative bacilli (76).

Gram-positive cocci (GPC) comprising catalase negative (173) and catalase positive (48).

Fermenters gram-negative bacilli (FGNB) comprising *Aeromonas*/*Vibrio* (22) and *Enterobacterales* (124).

#### Gram-positive bacilli (214)

*Actinomycetales*: 70 isolates of 29 species of the following 11 genera: *Actinomyces* sp., *Actinotignum s*p., *Cutibacterium* sp., *Gleimia* sp., *Gordonia* sp., *Nocardia* sp., *Rhodococcus* sp., *Schaalia* sp., *Streptomyces* sp., *Trueperella* sp., and *Tsukamurella* sp., were identified in VITEK MS PRIME and Microflex LT. The results are described in Table 3.

**TABLE 3:** Identification results of *Actinomycetales* (*n* = 70).

Correct identifications	VITEK MS PRIME	Microflex LT
Species/complex or group	51 (73%)	46 (66%)
Genus	8 (11%)	14 (20%)
No identification (NI)	11 (16%)	10 (14%)
NI with correct ID in RUO library	5/11 (45%)	N/A^*a*^
Total	70	70

^*a*^ N/A, not applied.

**Fig 2:**
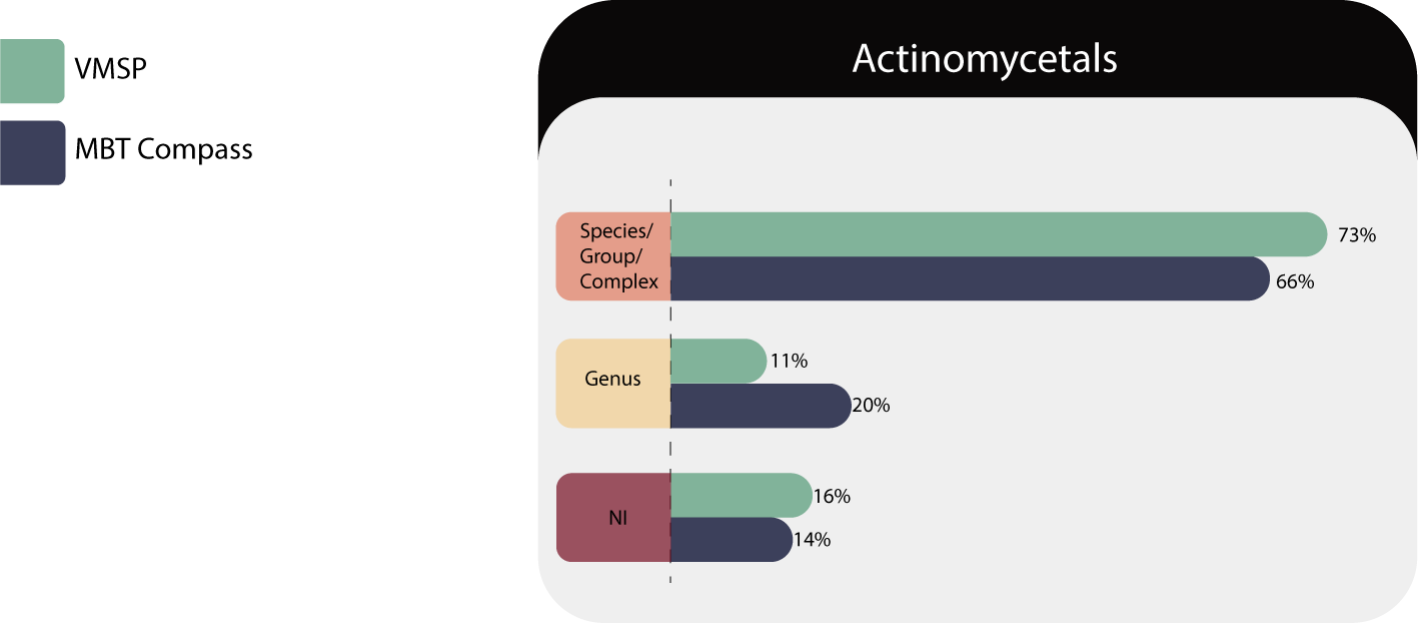
Identification performances for the *Actinomycetales* family.

As shown in Fig. 2, both platforms correctly identified most of the isolates, such as species of *Actinomyces* sp., *Actinotignum* sp., *Cutibacterium* sp., *Gordonia* sp., *Trueperella* sp., and *Nocardia* sp., but it is known that *Nocardia* species should be reported as complexes based on their antibiotic susceptibility profiles (27).

*Streptomyces* sp. and *Tsukamurella* sp. were evaluated at the genus level, because they require complex molecular characterization to arrive at the species level and the MS platforms usually identify them only at the genus level.

#### Limitations

VITEK MS PRIME did not identify two of the four isolates of *Schaalia* sp. despite its presence in the databases. *Nocardia pseudobrasiliensis* was not identified by any database and VITEK MS PRIME failed to identify *Nocardia wallacei* with the IVD library but we detected an improved identification with the SARAMIS RUO library.

On the other hand, in MBT COMPASS the differences were seen in score values lower than 2.0, which implied a greater number of identifications at the genus level, and no identifications of *Rhodococcus* because we could not achieve enough peaks with the direct extraction method tested; tube extraction methods are generally necessary for these morphologically difficult bacteria.

General recommendations for the *Actinomycetales* group and other dry rough colonies: based on our experience, we could suggest some simple additional steps as, the addition of a drop of water to the bacterial colony before picking it for MALDITOF or testing an *in-house* extraction protocol, developed by Rocca et al (28).

#### Sporulated GPB

Twenty-nine isolates of 11 species corresponding to eight genera (*Bacillus* sp., *Brevibacillus* sp., *Citobacillus* sp., *Heyndrickxia* sp., *Niallia* sp., *Paenibacillus* sp., *Priestia* sp., abd *Shouchella* sp.) were tested in both MS systems (Table 4).

**TABLE 4:** Identification results of sporulated GPB (*n* = 29) in both MS systems

Correct identifications	VITEK MS PRIME	Microflex LT
Species/complex or group	24 (83%)	24 (83%)
Genus	2 (7%)	4 (14%)
No identification (NI)	3 (10%)	1 (3%)
NI with correct ID in RUO library	1/3 (33%)	N/A
Total	29	29

Regarding the taxonomy, the members of *Bacillus* group have undergone an important update and these nomenclature changes, as *Priestia*, *Alkalihalobacillus*, and *Weizmannia*, are evident in the commercial libraries of the Bruker platform but not yet in VITEK MS PRIME (29).

**Fig 3:**
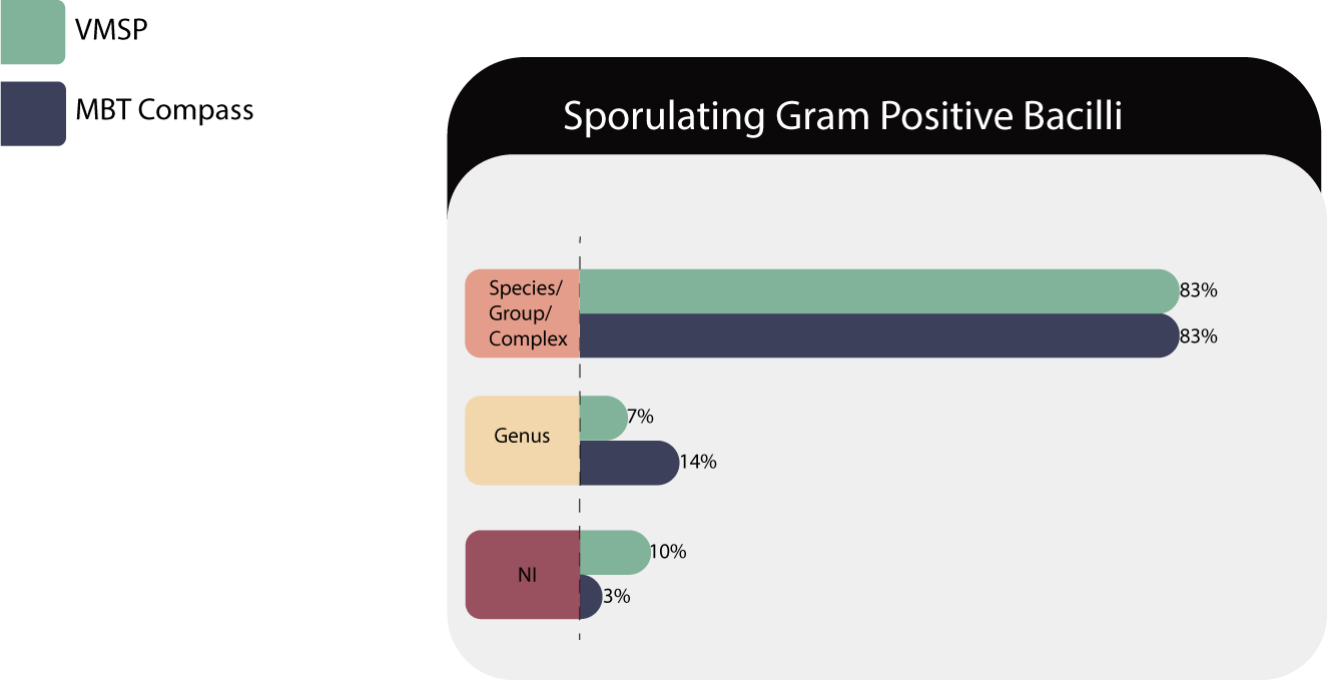
Identification performances for the SGPB family.

In Fig. 3, we can see that both platforms return multiple results (PRIME results) or without sufficient divergence between species (Bruker results), but those should be considered correct in both cases, because *Bacillus* should be mostly reported as groups, for example, those of the *Bacillus subtilis* group. In the vast majority of isolates corresponding to *Bacillus* genus, VITEK MS PRIME correctly identified at the group level as defined in the IVD database, as in the case of *Bacillus amyloliquefaciens/vallismortis/subtilis*.

As shown in Fig. 3, the differences observed in the percentages of identification at the genus level and NI level were due to the failure of VITEK MS PRIME to identify *Paenibacillus* sp., which was correctly identified at the genus level in Bruker’s platform.

#### Non-sporulating GPB

Fifty-five isolates of 18 species belonging to 12 genera (*Cellulosimicrobium* sp., *Curtobacterium* sp., *Dermabacter* sp., *Dolosigranulum s*p., *Erysipelothrix* sp., *Exiguobacterium* sp., *Gardnerella* sp., *Lacticaseibacillus* sp., *Lactobacillus* sp., *Listeria* sp., *Microbacterium* sp., and *Rothia* sp.) were tested in both MS systems (Table 5).

**TABLE 5:** Identification results of non-sporulating GPB (*n* = 55) in both MS systems

Correct identifications	VITEK MS PRIME	Microflex LT
Species/complex or group	51 (93%)	49 (89%)
Genus	4 (7%)	5 (9%)
No identification	0	1 (2%)
Total	55	55

We evaluated at the genus level the following microorganisms: *Curtobacterium* sp., *Exiguobacterium* sp., *Lactobacillus casei/paracasei/rhamnosus*, *acidophilus/gasseri*, and *Microbacterium* sp., because of their complexity.

On the other hand, it is important to emphasize that both platforms quickly resolved at the species level several microorganisms with clinical impact that are difficult to characterize in a conventional laboratory, such as *Dermabacter hominis*, *Dolosigranulum pigrum*, *Erysipelothrix rhusopathiae*, *Gardnerella vaginalis*, and *Rothia dentocariosa/mucilaginosa*.

**Fig 4:**
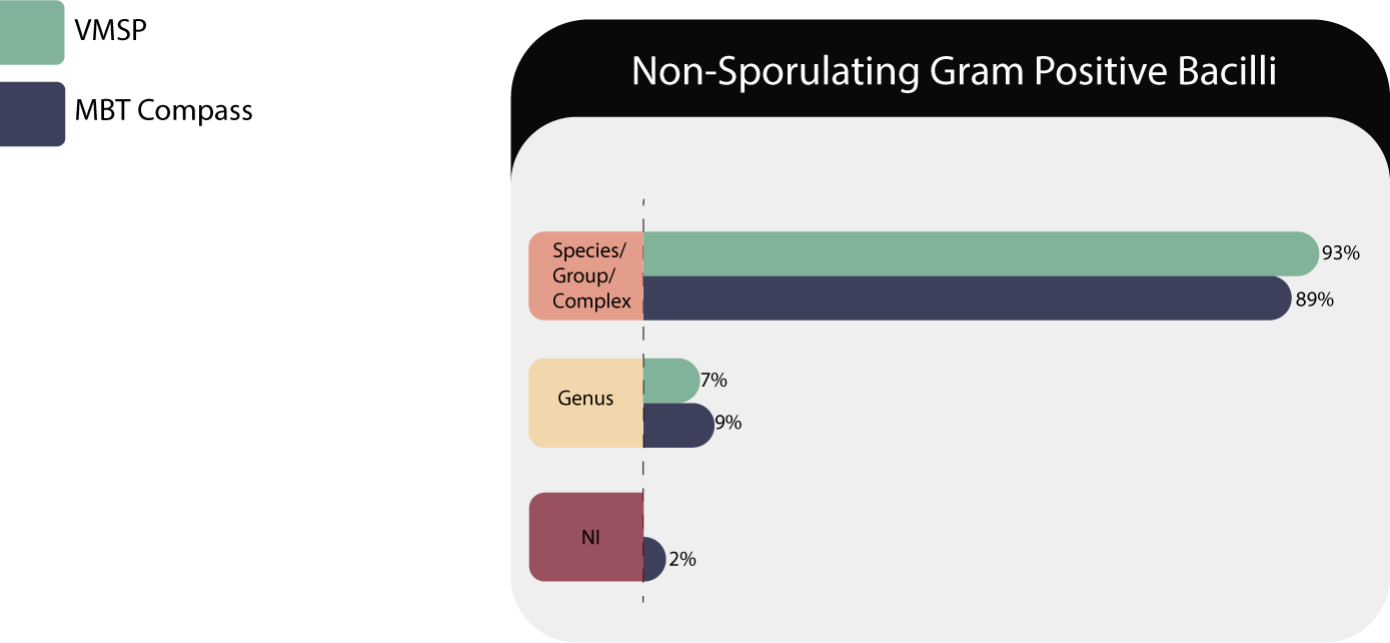
Identification performances for the non-sporulating GPB family.

As shown in Fig. 4*,* Microflex LT gave no identification in one case (2%) corresponding to *Rothia* sp. despite retesting it.

*Listeria monocytogenes* causes severe infection in the elderly, neonates, and the immunocompromised population with only a self-limited gastrointestinal infection in the immunocompetent. While *L. monocytogenes* is not the most common foodborne illness, it has the highest mortality rate secondary to its unique virulence factors (30).

In our experience**,** *L. monocytogenes* was never identified as another species in VITEK MS PRIME in none of the 14 isolates tested; however, Microflex LT did not achieve the discrimination to species level between *L. monocytogenes and Listeria innocua* with sufficient divergence. On the other hand, five strains of *L. innocua* tested yielded correct species identification on both platforms.

#### Coryneforms

Sixty isolates of 20 species/groups were tested in both MS systems and identification results are described in Table 6.

**TABLE 6:** Identification results of coryneforms (*n* = 60) in both MS systems

Correct identifications	VITEK MS PRIME	Microflex LT
Species/complex or group	49 (82%)	47 (78%)
Genus	3 (5%)	6 (10%)
No identification	8 (13%)	7 (12%)
Total	60	60

**Fig 5:**
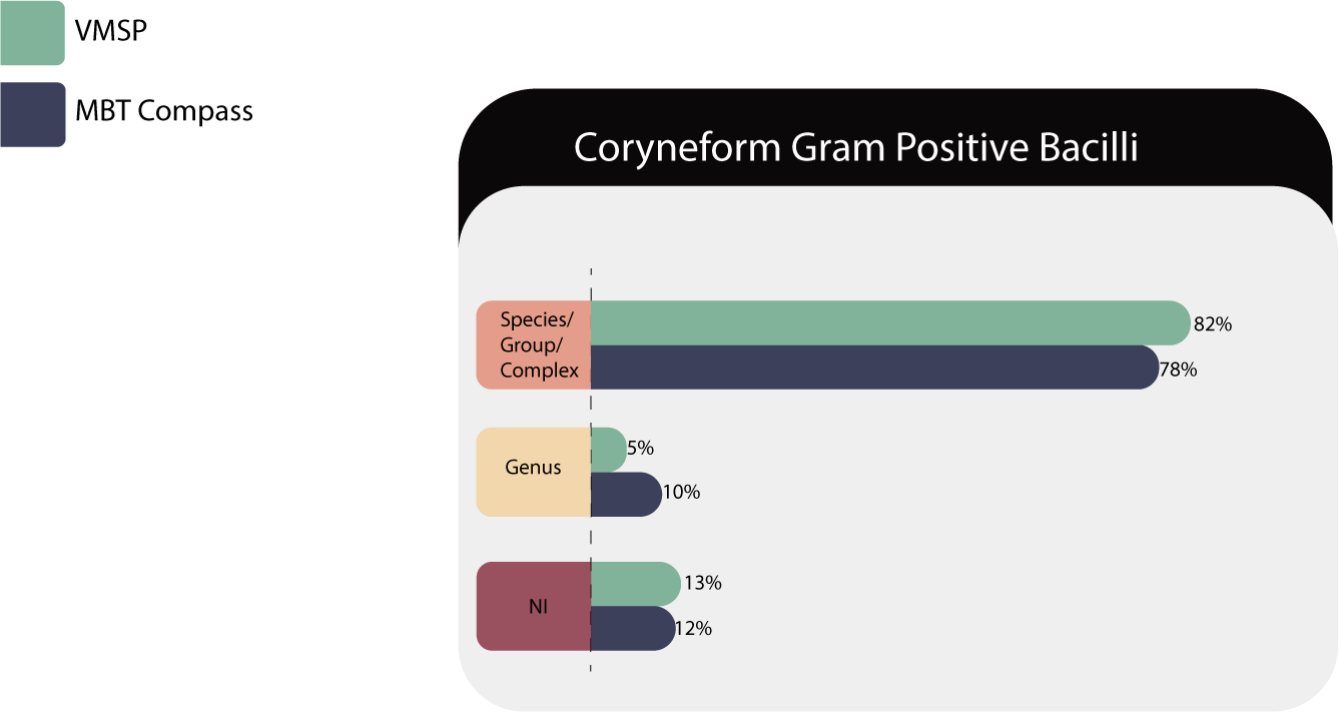
Identification performances for *Corynebacterium* sp.

As shown in Fig. 5, both platforms identified around 80% at the species level.

VITEK MS PRIME failed to identify *Corynebacterium riegelii (N* = 2) and *Corynebacterium variabile (N* = 2). Most of the isolates of *Corynebacterium kroppenstedtii* (three out of four) could not be identified by both IVD and RUO, despite being present in both databases. In our experience, the failure in the identification of *C. kroppenstedtii* by VITEK MS PRIME is a frequent limitation; that is, why we recommend to suspect this microorganism as the possible pathogen when we are dealing with a lipophilic isolate without a confident identification in the bioMérieux device.

On the other hand, MBT COMPASS library failed to identify *Corynebacterium coyleae (N* = 2) and *Corynebacterium freneyi (N* = 2).

Both platforms gave genus-level identifications for 10 isolates of *Corynebacterium mucifaciens*/*ureicelerivorans* and *Corynebacterium propinquum*/*Corynebacterium pseudodiphtheriticum* and neither gave reliable results for *C. variabile (N* = 2).

#### Non-fermenting gram-negative bacilli

Two hundred twenty-five isolates of 69 species/groups belonging to 30 genera (*Achromobacter* sp., *Acinetobacter* sp., *Agrobacterium* sp., *Alcaligenes* sp., *Bergeyella* sp., *Bordetella* sp., *Brucella* sp., *Burkholderia* sp., *Chromobacterium* sp., *Chryseobacterium* sp., *Comamonas* sp., *Cupriavidus* sp., *Delftia* sp., *Elizabethkingia* sp., *Empedobacter* sp., *Herbaspirillum* sp., *Inquilinus* sp., *Mannheimia* sp., *Moraxella* sp., *Myroides* sp., *Oligella* sp., *Pandoraea s*p., *Pseudomonas* sp., *Ralstonia* sp., *Roseomonas* sp., *Shewanella* sp., *Sphingobacterium* sp., *Sphingomonas* sp., *Stenotrophomonas* s*p.*, and *Weeksella* sp.) were tested in duplicate and the results are detailed in Table 7.

**TABLE 7:** Identification results of NFGNB (225) in both MS systems

Correct identifications	VITEK MS PRIME	Microflex LT
Species/complex or group	203 (90%)	190 (84%)
Genus	8 (4%)	31 (14%)
No identification (NI)	14 (6%)	4 (2%)
NI with correct ID in RUO library	12/14 (86%)	N/A^*a*^
Total	225	225

^*a*^ N/A, not applied.

**Fig 6:**
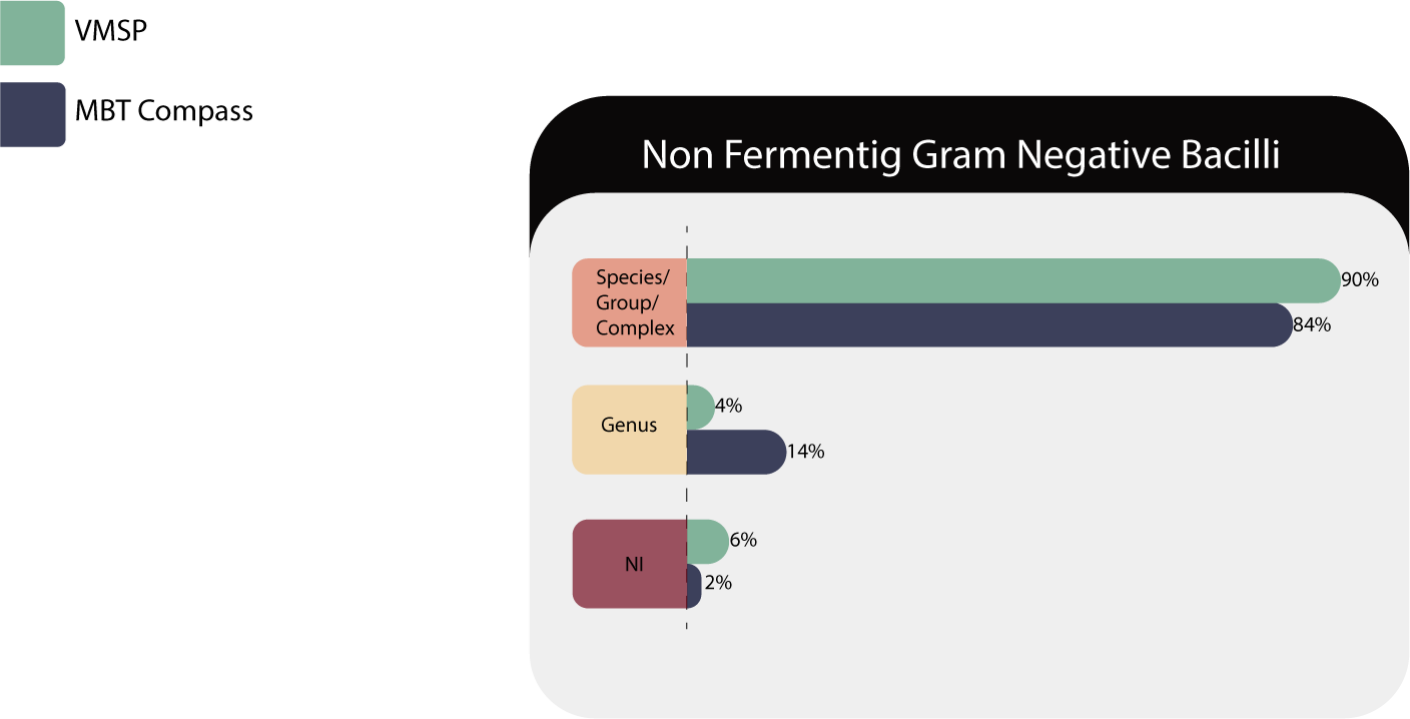
Identification performances for the NFGNB family.

Figure 6 shows that VITEK MS PRIME had a higher percentage of identifications at the species level (90% vs 84%), but when are taken into account the correct identifications at the genus level, Bruker achieved 98% while VITEK MS PRIME identified 94%.

On the other hand, in the group of 6% NI by the IVD database, 86% were resolved when comparing with the reference and super spectra present in the SARAMIS database of the RUO mode as in the following cases: *Myroides odoratus*, *Pseudomonas stutzeri*, despite its presence in the IVD database*, Pseudomonas otitidis*, which is only represented in the SARAMIS RUO database *and Comamonas kerstersii* only represented in SARAMIS at the genus level.

Both platforms correctly identified species of *Alcaligenes*, *Bergeyella*, *Bordetella*, *Chromobacterium*, *Chyseobacterium*, *Cupriavidus*, *Delftia*, *Elizabethkingia*, *Empedobacter*, *Ochrobactrum*, *Pandoraea*, *Pseudomonas*, *Ralstonia*, *Rhizobium*, *Roseomonas*, *Sphingomonas*, *Stenotrophomonas*, and *Weeksella*, important opportunistic pathogens difficult to characterize in the common clinical laboratory (31).

VITEK MS PRIME gave results of *A. xylosoxidans* 50%/*Achromobacter denitrificans* 50% while MBT Compass arrived at the genus level, giving results of *Achromobacter insolitus* or *Achromobacter mucicolens*, with score values below 2.0. The same situation happened with score values < 2.0 for *Acinetobacter pittii*, with two samples identified as *Acinetobacter lactucae*; although the rest of the *Acinetobacter s*pecies (A. *haemolyticus*, *A. junii*, *A. baumannii*, *A. lwoffii*, and *A. ursingii*) were correctly differentiated.

In this work, VITEK MS PRIME correctly identified *Burkholderia* species; this good performance did not occur in our first verification of VITEK MS in 2019, when the IVD KB 3.0 and the Saramis Premium V4.15.0 databases returned incorrect results for *Burkholderia seminalis*; clearly, this limitation was overcome in the new database.

On the other hand, the latest Bruker database requires molecular confirmation in the case of an identification of *B. cepacia, B. reimsis*, *B. seminalis*, and other species of *Burkholderia* sp. because usually different species with high-confidence results appear in the top 10 scores.

*Burkholderia cenocepacia* and *Burkholderia contaminans* are especially clinically relevant in cystic fibrosis patients in our country (32), and were identified at the species level by both platforms, avoiding the sequencing of the *rec*A gene and allowing differential diagnosis with other related species, such as *Pandoraea* sp., *Ralstonia* sp., or *Inquilinus limosus*. The last species mentioned did not give a confident result in VITEK MS PRIME, but only a single isolate was tested.

*Moraxella bovis*, *Moraxella lacunata,* and *Moraxella catarrhalis* were identified at the species level except *Moraxella bovoculi* that was identified at the genus level in Bruker and *Moraxella osloensis* in VITEK MS PRIME, which always appears as *Moraxella osloensis 50%/Enhydrobacter aerosaccus 50%*.

*Ochrobactrum anthropi* and *Ochrobactrum intermedium* were identified at the species level by both platforms, which are homotypic synonyms of *Brucella anthropi* and *Brucella intermedia*.

#### Fastidious gram-negative bacilli

Seventy-six isolates of 24 species/groups corresponding to 13 genera (*Actinobacillus* sp., *Aggregatibacter* sp., *Bacteroides* sp., *Campylobacter* sp., *Capnocytophaga* sp., *Cardiobacterium* sp., *Eikenella* sp., *Haemophilus* sp., *Histophilus* sp., *Kingella* sp., *Legionella* sp., *Neisseria s*p., and *Pasteurella* sp.) were evaluated in both MS systems and the results are described in Table 8.

**TABLE 8:** Identification results of fastidious gram-negative bacilli in both MS systems

Correct identifications	VITEK MS PRIME	Microflex LT
Species/complex or group	72 (95%)	72 (95%)
Genus	4 (5%)	4 (5%)
No identification (NI)	0	0
Total	76	76

Among the microorganisms with specific nutritional requirements, characterized by their slow growth and difficult characterization in the conventional laboratory, both platforms showed excellent performances with *Aggregatibacter*, *Campylobacter*, *Capnocytophaga*, *Cardiobacterium*, *Eikenella*, *Haemophilus influenzae* and *H. parainfluenzae*, *Histophilus somni*, *Pasteurella*, and *Legionella pneumophila/micdadei*. All of them identified at the species level from the initial bacterial culture, representing an important impact on the epidemiological and therapeutic aspects.

HACEK organisms are part of the normal flora of the mouth and upper respiratory tract in humans; that in certain favorable circumstances can cause a wide range of infections, of which infectious endocarditis is one of the most notable in people with underlying heart disease and people with artificial valves (33).

*Kingella kingae*, which usually colonizes the oropharynx and is one of the main pathogens involved in the development of bone infections and bacteremia in the pediatric population, was identified in minutes (34).

*Neisseria* sp. of clinical relevance such as *N. gonorrhoeae* and *N. meningitidis* were correctly identified at the species level (35).

Other species of *Neisseria*, that must be reported as a group, for example, *Neisseria sicca* group or *Neisseria flavescens* group was correctly identified.

**Fig 7:**
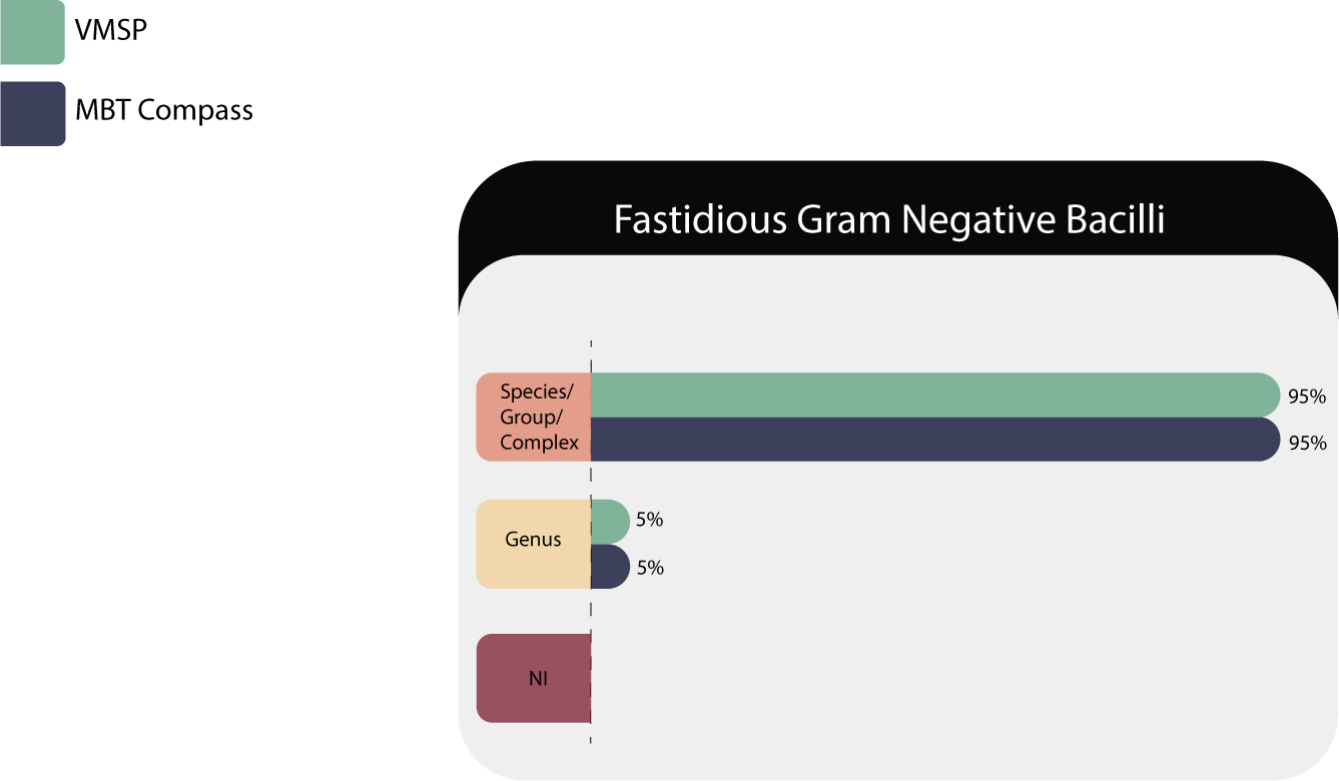
Identification performances for fastidious gram-negative bacilli family.

As shown in Fig. 7, the genus-level IDs were for *Actinobacillus lignieresii/pleuropneumoniae* and *Actinobacillus ureae/suis/equuli* that cannot be differentiated at species level by MALDI-TOF MS.

#### GPC catalase negative

One hundred seventy-three isolates of 41 species/group corresponding to 13 genera (*Abiotrophia* sp., *Aerococcus* sp., *Enterococcus* sp., *Facklamia* sp., *Gemella* sp., *Globicatella* sp., *Granulicatella* sp., *Helcococcus* sp., *Lactococcus* sp., *Leuconostoc s*p., *Pediococcus* sp., *Streptococcus* sp., and *Vagococcus* sp.) were evaluated in MALDI-TOF MS platforms and the results are described in Table 9.

**TABLE 9:** Identification results of gram-positive cocci (GPC) catalase negative (*n* = 173) in both MS systems

Correct identifications	VITEK MS PRIME	Microflex LT
Species/complex or group	168 (97%)	154 (89%)
Genus	3 (2%)	17 (10%)
No identification (NI)	2 (1%)	2(1%)
NI with correct ID in RUO library	2/2 (100%)	N/A^*a*^
Total	173	173

^*a*^ N/A, not applied.

**Fig 8:**
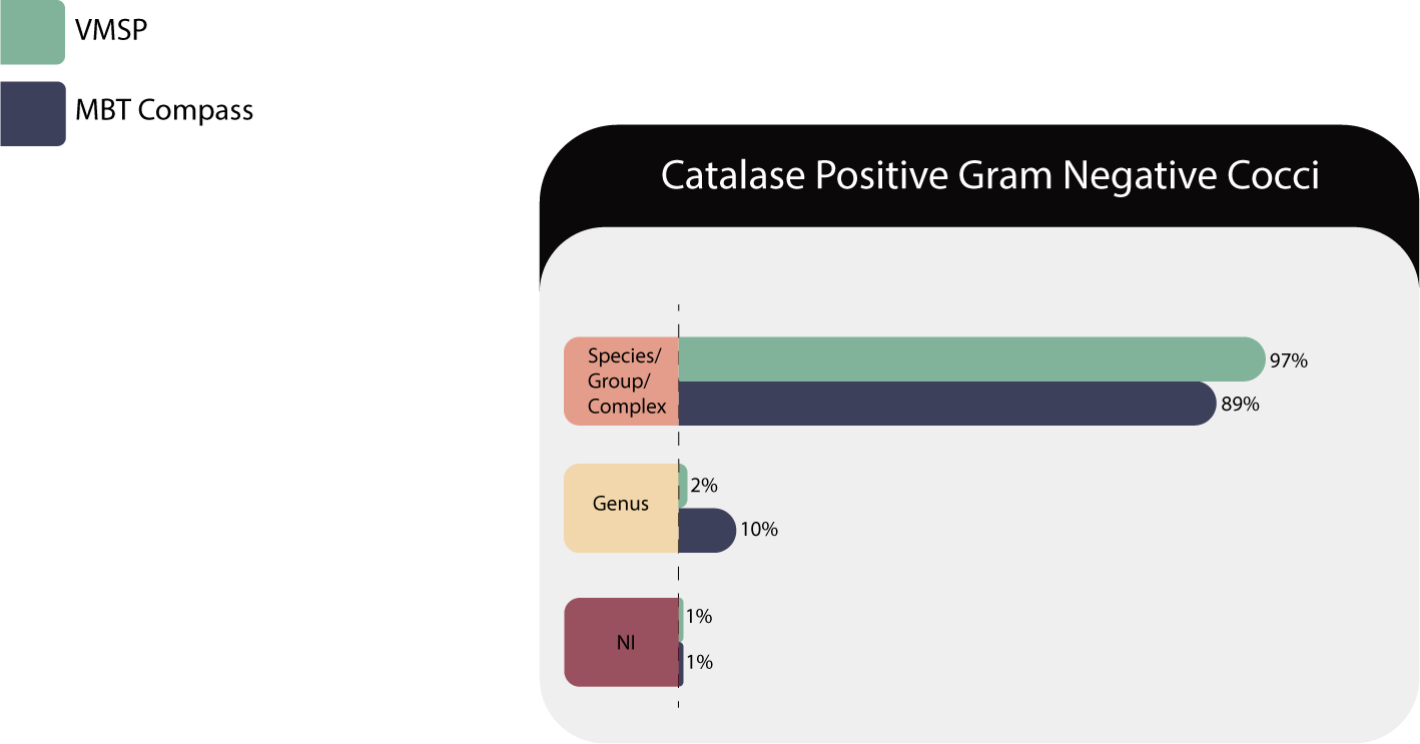
Identification performances for the GPC catalase negative family.

As shown in Fig. 8, identifications at the species level were achieved for *Abiotrophia*, *Enterococcus*, *Facklamia*, *Gemella*, *Helcococcus*, *Lactococcus*, *Leuconostoc*, *Pediococcus*, and *Vagococcus* genera that require molecular studies for complete identification (36).

For *Streptococcus* group, VITEK MS PRIME performed appropriately reporting the species of the *Streptococcus bovis* group, while in some cases, Microflex LT arrived to the correct group, but not to the correct species. This could be due to the settings for the spectrum generation in VITEK MS PRIME, which takes more time to process each well, with higher number of laser shots, granting greater diagnostic capacity in the gram positives. *Streptococcus pneumoniae* was correctly identified by both platforms, but *S. mitis/oralis* can appear as *S. pneumoniae* on Bruker with low score values.

*Streptococcus suis*, which presents greater pathogenic potential in Argentina, generally recovered from cerebrospinal fluid, was always identified at the species level, representing clinical impact in suspected patients in close contact with pigs or farm workers (37).

VITEK MS PRIME gave NI for one isolate of *Aerococcus christensenii* using the IVD database, but we obtained the correct identification in the RUO database. Bruker did not achieve identification of *Enterococcus mundtii* despite retesting.

#### GPC catalase positive

Forty-eight isolates of 15 species/group from four genera (*Dermacoccus* sp., *Mammaliicoccus s*p., *Micrococcus* sp., and *Staphylococcus* sp.) were tested and results are described in Table 10.

**TABLE 10:** Identification results of gram-positive cocci (GPC) catalase positive in both MS systems

Correct identifications	VITEK MS PRIME	Microflex LT
Species/complex or group	46 (96%)	46 (96%)
Genus	1 (2%)	2 (4%)
No identification (NI)	1 (2%)	0
NI with correct ID in RUO library	1/1 (100%)	N/A^*a*^
Total	48	48

^*a*^ N/A, not applied.

**Fig 9:**
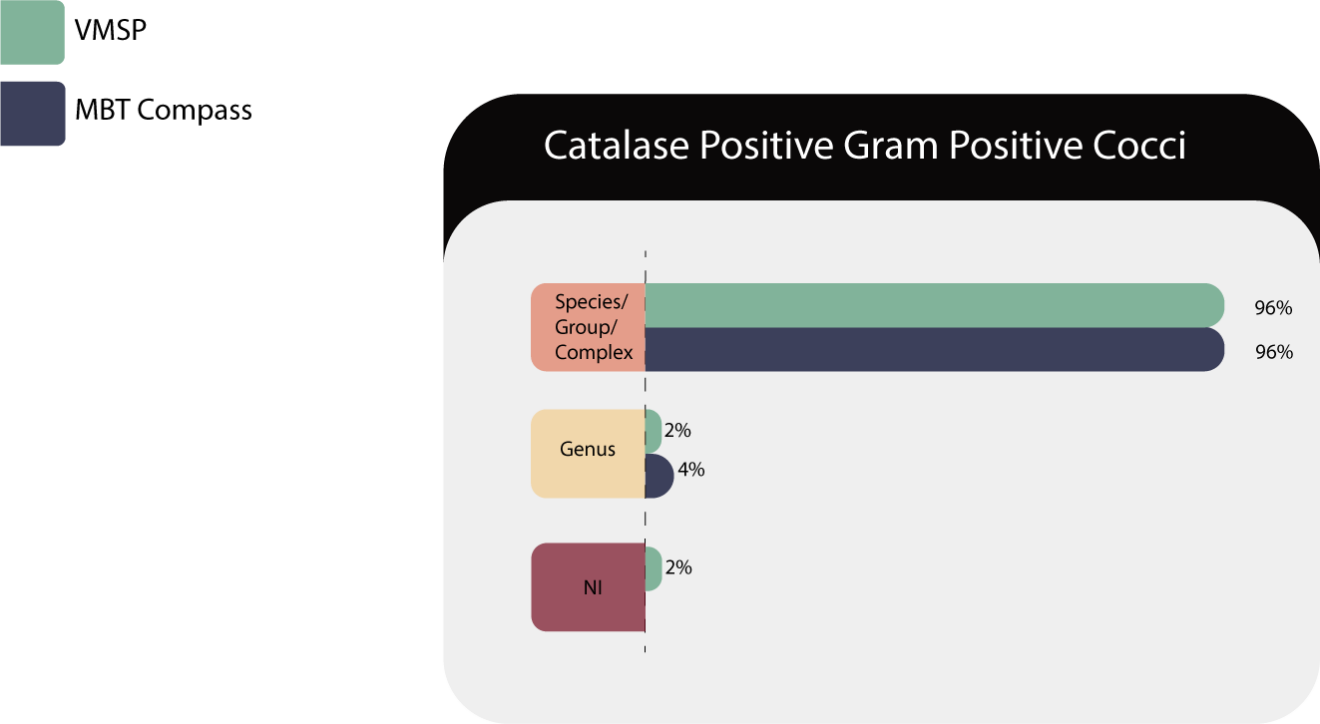
Identification for the gram-positive cocci (GPC) catalase positive.

VITEK MS PRIME failed to identify *Micrococcus luteus* (Fig. 9). One of the two samples of *Staphylococcus pettenkoferi* could not be identified by the IVD database but was identified using the RUO database. Both platforms correctly identified *Staphylococcus aureus* and differentiated it from other coagulase-negative species, important nosocomial pathogens with associated resistance mechanisms (38). *Staphylococcus pseudintermedius/intermedius* are not discriminable at the species level through MS and should be reported as *Staphylococcus intermedius* group.

#### Fermenters gram-negative bacilli

*Aeromonas* and *Vibrio* comprising 22 isolates (*A. caviae* complex, *A. hydrophila* complex, *Aeromonas veronii* complex, *and Vibrio cholerae*) were evaluated in both MS systems and the results are described in Table 11.

**TABLE 11:** Identification results of *Aeromonas* and *Vibrio* (*n* = 22) in both MS systems

Correct identifications	VITEK MS PRIME	Microflex LT
Species/complex or group	20 (91%)	17 (77%)
Genus	2 (9%)	5 (23%)
No identification (NI)	0	0
Total	22	22

**Fig 10:**
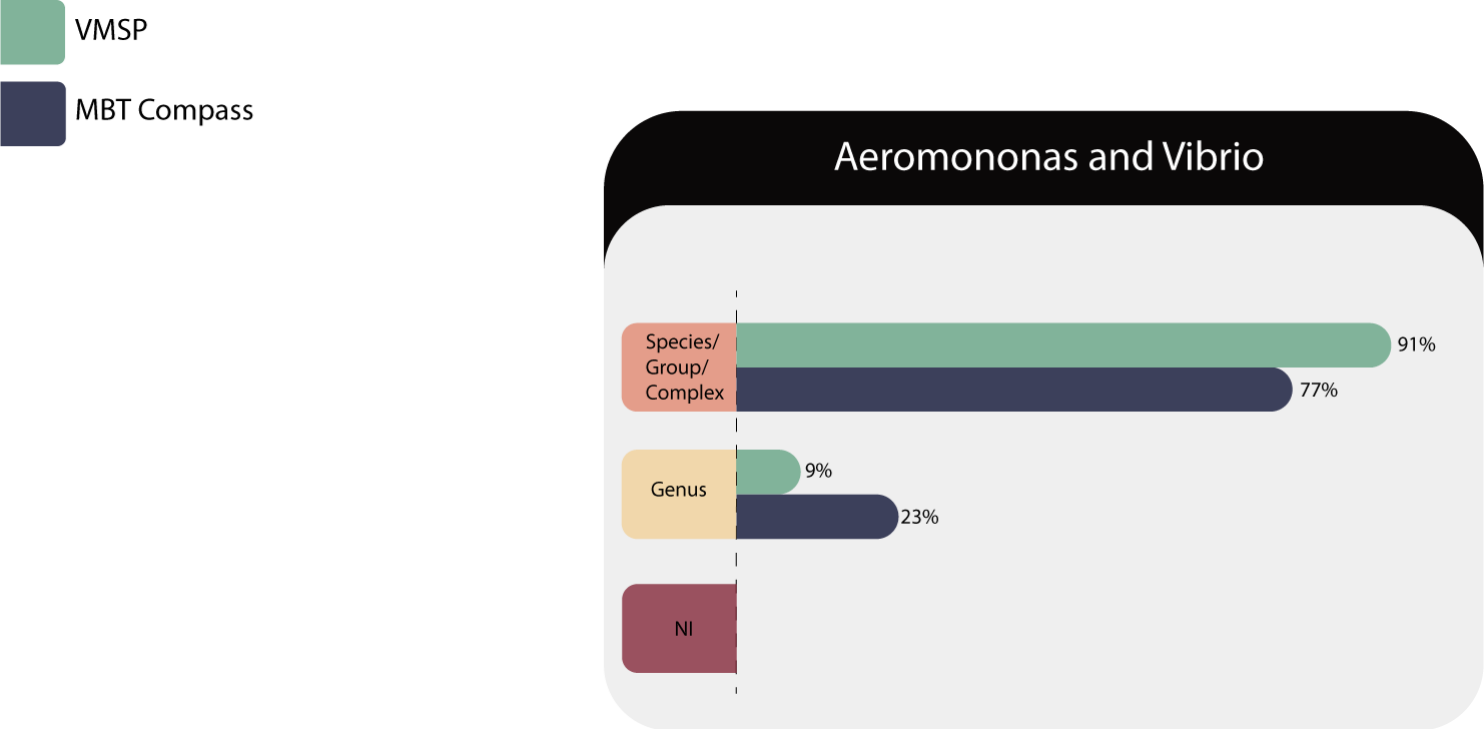
Identification performances for *Aeromonas* and *Vibrio*.

Both platforms identified *Vibrio cholerae* at the species level. In the case of *Aeromonas*, VITEK MS PRIME could not discriminate the correct complex in two opportunities, while Compass library failed on five isolates (Fig. 10).

In our experience, Bruker’s platform can not differentiate *A. caviae* from A. *hydrophila*.

The IVD database from VMSP reported the *Aeromonas veronii* complex (*veronii* biovar *sobria*) as *Aeromonas sobria* 50% and *Aeromonas veronii* 50%.

However, the other platform also does not report the species of the complex with sufficient divergence, usually reporting *A. veronii/A. ichthiosmia* (39).

Both platforms detected correctly the species of the *A. hydrophila* complex, as *A. hydrophila*, *A. bestiarum*, and *A. salmonicida*.

### Enterobacterales

One hundred twenty-four isolates of 24 species/group corresponding to 15 genera (*Citrobacter* sp.*, Cronobacter* sp., *Edwardsiella* sp., *Enterobacter* sp., *Escherichia* sp., *Klebsiella* sp., *Leclercia* sp., *Lelliottia* sp., *Morganella s*p., *Pantoea* sp., *Plesiomonas* sp., *Proteus* sp., *Providencia s*p., *Salmonella* sp., and *Serratia* sp.) were tested in both systems and the results are described in Table 12.

**TABLE 12:** Identification results of *Enterobacterales* (*n* = 124) in both systems

Correct identifications	VITEK MS PRIME	Microflex LT
Species/complex or group	123 (99%)	121 (98%)
Genus	0	3 (2%)
No identification (NI)	1 (1%)	0
Total	124	124

**Fig 11:**
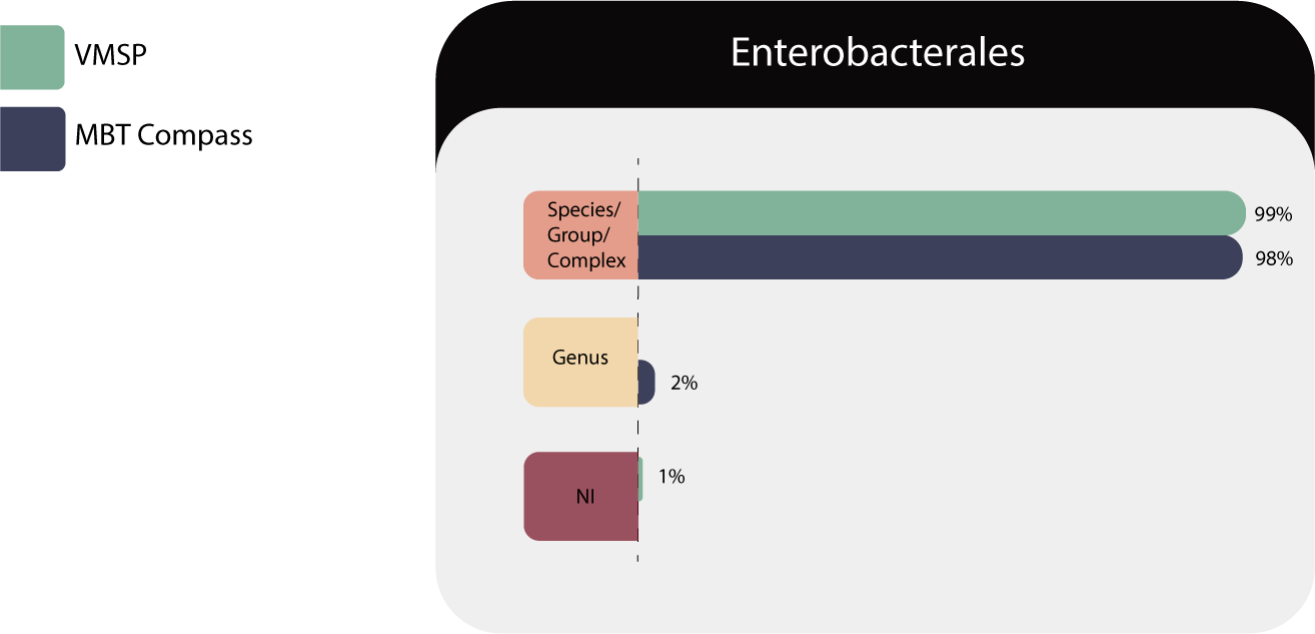
Identification performances for the *Enterobacterales* family.

*Enterobacterales* were all identified at the species/group level considering that according to the NCBI taxonomy browser, they should be reported as complexes, including *C. freundii*, *C. braakii*, *C. gillenii*, *C. murliniae*, *C. rodentium*, *C. werkmanii*, *C. youngae*, *C. koseri*, and *C. farmeri*, all species of the *Citrobacter freundii* complex (see Fig. 11).

The same happens with *Klebsiella pneumoniae* complex (*K. pneumoniae/variicola*) and *E. cloacae* complex, which is reported in VMSP as *E. cloacae* (50%)/*Enterobacter asburiae* (50%) or *E. cloacae* (33.3%)/*Enterobacter asburiae* (33.3%)/*Enterobacter hormaechei* (33.3%), while MBT Compass showed green scores without sufficient divergence for different species, such as *E. hormaechei*, *E. bugandensis*, *E. ludwigii*, and *E. roggenkampii*.

Both platforms arrived properly at the species level for *Cronobacter sakazakii*, *Edwardsiella tarda*, *Leclercia adecarboxylata*, *Morganella*, *Providencia*, *Plesiomonas*, *Raoultella*, and *Serratia*.

Furthermore, we reaffirm the intrinsic limitation of the methodology regarding the impossibility of discriminating *E. coli* from *Shigella* sp. and also discrimination of *Salmonella* serovars; despite the fact that several groups are currently making important progress in the differentiation of STEC O157:H7 strains through rapid detection of biomarkers in MALDI-TOF MS (40).

### Limitations

VITEK MS PRIME did not identify the only isolate of *Lelliottia amnigena* that was tested. For Bruker Biotyper, one of the three samples of *Proteus vulgaris* was misidentified as *Proteus hauseri* and one of two *Pantoea agglomerans* was misidentified as *Pantoea anthophila*.

## DISCUSSION

Recently, the new VITEK MS PRIME was introduced in Argentina. The reference laboratory of the National Institute of Infectious Diseases, acting as the coordinator of ReNaEM, completed a verification process following the CLSI M58 guideline, with results indicating important outcomes. Following the analysis of 882 bacterial isolates characterized utilizing polyphasic taxonomy methods (including biochemical tests, gene sequencing, and whole-genome sequencing in specific cases), the evaluation of VITEK MS PRIME in conjunction with IVD v3.2 and SARAMIS v4.17 databases and Microflex LT with the MBT Compass Explorer Database yielded accurate genus and species levels identification in few minutes.

VITEK MS PRIME achieved correct species identification in 91% of the isolates, while MBT Compass Explorer in 87% of them; this may be due to the fact that the latter is a device with more than10 years of sustained use which has reduced the quality of the spectrum and therefore the score values arising from the comparisons, which, according to the manufacturer, should be considered correct just at the genus level. As the functionality of the instruments themselves played a major role in the generation of the data presented, this fact represents a major limitation of the study.

Another limitation of the study is the comparison between IVD and RUO libraries; Vitek IVD V3.2 library is FDA cleared; however, the comparator database MBT Compass Explore v12 used on the Microflex LT System is RUO.

The use of the RUO SARAMIS library facilitated identifications for previously unknown cases. Therefore, it is valid to recommend the use of the RUO mode for the NI with the IVD database since in our experience, it resolved more than half of the NI and in the new platform, the procedure is much easier, not requiring a second acquisition of the samples.

Noteworthy is the system’s demonstrated accuracy comparable to whole-genome sequencing, especially evident in the case of the *Streptococcus bovis* group. Overall, the improved accuracy resulted in minimized errors, expedited diagnoses, and more efficient resource utilization.

For its part, MBT Compass presented better performance among non-fermenting bacilli, which may be due to its more complete databases.

Both MS systems reliably identified fastidious species allowing for the establishment of specific and timely appropriate antimicrobial therapy.

They manage to discriminate complex pathogens that are difficult to characterize and are of emerging importance in the hospital setting.

They have the same or better resolution as rRNA gene sequencing but with much cheaper and faster results.

Each has its advantages and limitations, so it will be up to each individual laboratory to evaluate which one is most appropriate for its workflow. Once purchased, both platforms represent enormous cost savings in supplies and labor in any laboratory.

These studies yielded invaluable information that needed to be shared with clinical laboratories incorporating MALDI-TOF MS technology. To achieve this, the website http://www.anlis.gov.ar/renaem/ was designed, with the primary objective of disseminating the generated information. This is a free platform easily accessible from any smart electronic device; it is dynamic and continually undergoing improvement and updates. This website includes a description of the network structure, manufacturer-recommended protocols, a standard operating procedure manual, updates, news, and a list of publications by network participants.

Additionally, a manual for interpreting MALDI-TOF MS results, was published. The contents include information on suggested molecular methods for reference identification and algorithms and tables with phenotypic markers and their limitations; different sample preparation techniques; recommendations for interpreting identification results; and newly accepted and validated score values by National Reference Laboratories. The open access to this Manual, along with its availability through the Knowledge Management System (http://sgc.anlis.gob.ar/), allows clinical laboratories to have confidence that the information obtained with this new technology is reliable and validated by national reference laboratories.

This type of study is valuable for providing recommendations to laboratories looking to incorporate this new platform and wishing to become part of RENAEM.

### Transforming clinical microbiology in Argentina through collaboration and innovation

In conclusion, the establishment and growth of RENAEM represent a significant milestone in Argentina’s clinical microbiology landscape. The collaborative synergy among clinical laboratories, research groups, and healthcare institutions has not only successfully integrated MALDI-TOF MS technology into routine diagnostics but has also driven continuous progress in database expansion, validation, and method standardization. RENAEM’s dedication to knowledge sharing through workshops, publications, and an extensive online platform highlights its commitment to championing excellence in microbiological diagnosis. As the pioneering force responsible for implementing the first nationwide MS network, RENAEM exemplifies how collaboration and innovation can transform public health initiatives.

## References

[1] Sampedro A, Ceballos Mendiola J, Aliaga Martínez L. 2018. Chapter Three - MALDI-TOF commercial platforms for bacterial identification, p 47–57. In Cobo F, the use of mass spectrometry technology (MALDI-TOF) in clinical microbiology. Academic Press.

[2] Jamal W, Albert MJ, Rotimi VO. 2014. Real-time comparative evaluation of bioMerieux VITEK MS versus Bruker Microflex MS, two matrix-assisted laser desorption-ionization time-of-flight mass spectrometry systems, for identification of clinically significant bacteria. BMC Microbiol 14:289. doi:10.1186/s12866-014-0289-0 25433488 PMC4290442

[3] Lee M, Chung HS, Moon HW, Lee SH, Lee K. 2015. Comparative evaluation of two matrix-assisted laser desorption ionization time-of-flight mass spectrometry (MALDI-TOF MS) systems, Vitek MS and Microflex LT, for the identification of Gram-positive cocci routinely isolated in clinical microbiology laboratories. J Microbiol Methods 113:13–15. doi:10.1016/j.mimet.2015.03.020 25818760

[4] Porte L, García P, Braun S, Ulloa MT, Lafourcade M, Montaña A, Miranda C, Acosta-Jamett G, Weitzel T. 2017. Head-to-head comparison of Microflex LT and Vitek MS systems for routine identification of microorganisms by MALDI-TOF mass spectrometry in Chile. PLoS One 12:e0177929. doi:10.1371/journal.pone.0177929 28542393 PMC5436840

[5] Rocca MF, Barrios R, Zintgraff J, Martínez C, Irazu L, Vay C, Prieto M. 2019. Utility of platforms Viteks MS and Microflex LT for the identification of complex clinical isolates that require molecular methods for their taxonomic classification. PLoS One 14:e0218077. doi:10.1371/journal.pone.0218077 31269022 PMC6608940

[6] FDA. 1998. Available from: http://www.accessdata.fda.gov/cdrh_docs/reviews/K124067.pdf

[7] FDA. 1998. Available from: http://www.accessdata.fda.gov/cdrh_docs/reviews/K130831.pdf

[8] Rocca MF, Almuzara M, Barberis C, Vay C, Viñes P, Prieto M. 2020. Presentación del sitio web de la red Nacional de identificación microbiológica por espectrometría de masas. manual para la interpretación de resultados de MALDI-TOF MS. Rev Arg Microbiol 52:83–84. doi:10.1016/j.ram.2019.03.001 31178239

[9] CLSI. 2017. Methods for the identification of cultured microorganisms using matrix-assisted laser desorption/ionization time-of-flight mass spectrometry. In CLSI guideline M58, 1st ed. Clinical and Laboratory Standards Institute, Wayne, PA.

[10] Almuzara M, Barberis C, Traglia G, Famiglietti A, Ramirez MS, Vay C. 2015. Evaluation of matrix-assisted laser desorption ionization-time-of-flight mass spectrometry for species identification of nonfermenting Gram-negative bacilli. J Microbiol Methods 112:24–27. doi:10.1016/j.mimet.2015.03.004 25765149

[11] Barberis C, Almuzara M, Join-Lambert O, Ramírez MS, Famiglietti A, Vay C. 2014. Comparison of the Bruker MALDI-TOF mass spectrometry system and conventional phenotypic methods for identification of gram-positive rods. PLoS One 9:e106303. doi:10.1371/journal.pone.0106303 25184254 PMC4153636

[12] Almuzara M, Barberis C, Velázquez VR, Ramirez MS, Famiglietti A, Vay C. 2016. Matrix-assisted laser desorption ionization-time-of-flight mass spectrometry (MALDI-TOF MS) as a reliable tool to identify species of catalase-negative gram-positive cocci not belonging to the Streptococcus Genus. Open Microbiol J 10:202–208. doi:10.2174/1874285801610010202 28217192 PMC5278551

[13] Barberis C, Ledesma M, Álvarez C, Famiglietti A, Almuzara M, Vay C. 2021. Analysis of the diversity of Actinomyces/Actinotignum clinical isolates in a university hospital. Rev Argent Microbiol 53:202–209. doi:10.1016/j.ram.2020.11.005 33402284

[14] Bizzini A, Durussel C, Bille J, Greub G, Prod’hom G. 2010. Performance of matrix-assisted laser desorption ionization-time of flight mass spectrometry for identification of bacterial strains routinely isolated in a clinical microbiology laboratory. J Clin Microbiol 48:1549–1554. doi:10.1128/JCM.01794-09 20220166 PMC2863943

[15] van Veen SQ, Claas ECJ, Kuijper EJ. 2010. High-throughput identification of bacteria and yeast by matrix-assisted laser desorption ionization-time of flight mass spectrometry in conventional medical microbiology laboratories. J Clin Microbiol 48:900–907. doi:10.1128/JCM.02071-09 20053859 PMC2832429

[16] Mellmann A, Cloud J, Maier T, Keckevoet U, Ramminger I, Iwen P, Dunn J, Hall G, Wilson D, Lasala P, Kostrzewa M, Harmsen D. 2008. Evaluation of matrix-assisted laser desorption ionization-time-of-flight mass spectrometry in comparison to 16S rRNA gene sequencing for species identification of nonfermenting bacteria. J Clin Microbiol 46:1946–1954. doi:10.1128/JCM.00157-08 18400920 PMC2446840

[17] Topić Popović N, Kazazić SP, Bojanić K, Strunjak‐Perović I, Čož‐Rakovac R. 2023. Sample preparation and culture condition effects on MALDI‐TOF MS identification of bacteria: a review. Mass Spectrom Rev 42:1589–1603. doi:10.1002/mas.21739 34642960

[18] Petti C, Bosshard P, Brandt M, Clarridge J, Feldblyum T, Foxall P, Furtado M, Pace N, Procop G. 2008. Interpretive criteria for identification of bacteria and fungi by DNA target sequencing; CLSI approved guideline

[19] Ibrahim A, Gerner-Smidt P, Liesack W. 1997. Phylogenetic relationship of the twenty-one DNA groups of the genus Acinetobacter as revealed by 16S ribosomal DNA sequence analysis. Int J Syst Bacteriol 47:837–841. doi:10.1099/00207713-47-3-837 9226915

[20] Mahenthiralingam E, Bischof J, Byrne SK, Radomski C, Davies JE, Av-Gay Y, Vandamme P. 2000. DNA-based diagnostic approaches for identification of Burkholderia cepacia complex, Burkholderia vietnamiensis, Burkholderia multivorans, Burkholderia stabilis, and Burkholderia cepacia Genomovars I and III. J Clin Microbiol 38:3165–3173. doi:10.1128/JCM.38.9.3165-3173.2000 10970351 PMC87345

[21] Khamis A, Raoult D, La Scola B. 2004. rpoB gene sequencing for identification of Corynebacterium species. J Clin Microbiol 42:3925–3931. doi:10.1128/JCM.42.9.3925-3931.2004 15364970 PMC516356

[22] Seung Bok H, Kyudong H, Bo Ra S, Kyeong Seob S, Byeong Cheol R. 2012. First case of Nocardia nova spinal abscess in an immunocompetent patient. Braz J Infect Dis 16:196–199. doi:10.1590/S141386702012000200017 22552466

[23] Drevinek M, Dresler J, Klimentova J, Pisa L, Hubalek M. 2012. Evaluation of sample preparation methods for MALDI-TOF MS identification of highly dangerous bacteria. Lett Appl Microbiol 55:40–46. doi:10.1111/j.1472-765X.2012.03255.x 22512320

[24] Fernández-Bravo A, Figueras MJ. 2020. An update on the genus Aeromonas: taxonomy, epidemiology, and pathogenicity. Microorganisms 8:129. doi:10.3390/microorganisms8010129 31963469 PMC7022790

[25] Zheng W, Tan TK, Paterson IC, Mutha NVR, Siow CC, Tan SY, Old LA, Jakubovics NS, Choo SW. 2016. StreptoBase: an oral Streptococcus mitis group genomic resource and analysis platform. PLoS ONE 11:e0151908. doi:10.1371/journal.pone.0151908 27138013 PMC4854451

[26] Annavajhala MK, Gomez-Simmonds A, Uhlemann AC. 2019. Multidrug-resistant Enterobacter cloacae complex emerging as a global, diversifying threat. Front Microbiol 10:44. doi:10.3389/fmicb.2019.00044 30766518 PMC6365427

[27] Lebeaux D, Bergeron E, Berthet J, Djadi-Prat J, Mouniée D, Boiron P, Lortholary O, Rodriguez-Nava V. 2019. Antibiotic susceptibility testing and species identification of Nocardia isolates: a retrospective analysis of data from a French expert laboratory, 2010-2015. Clin Microbiol Infect 25:489–495. doi:10.1016/j.cmi.2018.06.013 29933049

[28] Rocca MF, Zintgraff JC, Vay C, Prieto M. 2023. Optimizing main spectra profiles for development of customer databases by using a small heat shock in the standard MALDI-TOF MS protocol. Int J Mass Spectrom 489:117065. doi:10.1016/j.ijms.2023.117065

[29] Gupta RS, Radhey S, Patel S, Saini N, Chen S. 2020. Robust demarcation of 17 distinct Bacillus species clades, proposed as novel Bacillaceae genera, by phylogenomics and comparative genomic analyses: description of Robertmurraya kyonggiensis sp. nov. and proposal for an emended genus Bacillus limiting it only to the members of the Subtilis and Cereus clades of species. Int J Syst Evol Microbiol 70:5753–5798. doi:10.1099/ijsem.0.004475 33112222

[30] Rogalla D, Bomar PA. 2023. Listeria monocytogenes. In StatPearls [Internet]. StatPearls Publishing, Treasure Island (FL). 30521259

[31] Slenker AK, Hess BD, Jungkind DL, DeSimone JA. 2012. Fatal case of Weeksella virosa sepsis. J Clin Microbiol 50:4166–4167. doi:10.1128/JCM.01761-12 23035202 PMC3503020

[32] Cipolla L, Rocca MF, Martinez C, Aguerre L, Barrios R, Prieto M. 2018. Prevalencia de especies del complejo Burkholderia cepacia en pacientes con fibrosis quística en Argentina durante el período 2011-2015. Enferm Infecc Microbiol Clín 36:431–434. doi:10.1016/j.eimc.2017.09.002 29055510

[33] Khaledi M, Sameni F, Afkhami H, Hemmati J, Asareh Zadegan Dezfuli A, Sanae MJ, Validi M. 2022. Infective endocarditis by HACEK: a review. J Cardiothorac Surg 17:185. doi:10.1186/s13019-022-01932-5 35986339 PMC9389832

[34] Basmaci R, Ilharreborde B, Bidet P, Doit C, Lorrot M, Mazda K, Bingen E, Bonacorsi S. 2012. Isolation of Kingella kingae in the oropharynx during K. kingae arthritis in children. Clin Microbiol Infect: Offl Pub European Soc Clin Microbiol Infect Dis 18:E134–6. doi:10.1111/j.1469-0691.2012.03799.x 22390653

[35] Suay-García B, Pérez-Gracia MT. 2020. Neisseria gonorrhoeae Infections. Pathogens 9:647. doi:10.3390/pathogens9080647 32806522 PMC7459638

[36] Rahmati E, Martin V, Wong D, Sattler F, Petterson J, Ward P, Butler-Wu SM, She RC. 2017. Facklamia species as an underrecognized pathogen. Open Forum Infect Dis 4:36. doi:10.1093/ofid/ofw272 PMC5414014 28480264

[37] Callejo R, Zheng H, Du P, Prieto M, Xu J, Zielinski G, Auger JP, Gottschalk M. 2016. Streptococcus suis serotype 2 strains isolated in Argentina (South America) are different from those recovered in North America and present a higher risk for humans. JMM Case Rep 3:e005066. doi:10.1099/jmmcr.0.005066 28348788 PMC5343146

[38] Obolski U, Alon D, Hadany L, Stein GY. 2014. Resistance profiles of coagulase-negative staphylococci contaminating blood cultures predict pathogen resistance and patient mortality. J Antimicrob Chemother 69:2541–2546. doi:10.1093/jac/dku156 24855122

[39] Collins MD, Martinez-Murcia AJ, Cai J. 1993. Aeromonas enteropelogenes and Aeromonas ichthiosmia are identical to Aeromonas trota and Aeromonas veronii, respectively, as revealed by small-subunit rRNA sequence analysis. Int J Syst Bacteriol 43:855–856. doi:10.1099/00207713-43-4-855 8240968

[40] Manfredi E, Rocca MF, Zintgraff J, Irazu L, Miliwebsky E, Carbonari C, Deza N, Prieto M, Chinen I. 2023. Rapid and accurate detection of Shiga toxin-producing Escherichia coli (STEC) serotype O157: H7 by mass spectrometry directly from the isolate, using 10 potential biomarker peaks and machine learning predictive models. J Med Microbiol 72. doi:10.1099/jmm.0.001675 37130048

